# Low heat tolerance and high desiccation resistance in nocturnal bees and the implications for nocturnal pollination under climate change

**DOI:** 10.1038/s41598-023-49815-6

**Published:** 2023-12-15

**Authors:** Victor H. Gonzalez, Rachel Manweiler, Adam R. Smith, Kennan Oyen, David Cardona, William T. Wcislo

**Affiliations:** 1 Department of Ecology and Evolutionary Biology, University of Kansas, Lawrence, KS 66045 USA; 2 Department of Biological Sciences, George Washington University, Washington, District of Columbia USA; 3 Animal Disease Research Unit, Agricultural Research Service, United States Department of Agriculture, Pullman, WA 99164 USA; 4 Smithsonian Tropical Research Institute, Panama, Republic of Panama

**Keywords:** Ecology, Climate-change ecology, Ecophysiology, Tropical ecology

## Abstract

Predicting insect responses to climate change is essential for preserving ecosystem services and biodiversity. Due to high daytime temperatures and low humidity levels, nocturnal insects are expected to have lower heat and desiccation tolerance compared to diurnal species. We estimated the lower (CT_Min_) and upper (CT_Max_) thermal limits of *Megalopta*, a group of neotropical, forest-dwelling bees. We calculated warming tolerance (WT) as a metric to assess vulnerability to global warming and measured survival rates during simulated heatwaves and desiccation stress events. We also assessed the impact of body size and reproductive status (ovary area) on bees’ thermal limits. *Megalopta* displayed lower CT_Min_, CT_Max_, and WTs than diurnal bees (stingless bees, orchid bees, and carpenter bees), but exhibited similar mortality during simulated heatwave and higher desiccation tolerance. CT_Min_ increased with increasing body size across all bees but decreased with increasing body size and ovary area in *Megalopta*, suggesting a reproductive cost or differences in thermal environments. CT_Max_ did not increase with increasing body size or ovary area. These results indicate a greater sensitivity of *Megalopta* to temperature than humidity and reinforce the idea that nocturnal insects are thermally constrained, which might threaten pollination services in nocturnal contexts during global warming.

## Introduction

Bees are the most important pollinators of wild and cultivated plants. While the importance of bees in diurnal pollination is undeniable, their role in nocturnal pollination has largely been ignored or unappreciated, perhaps because of the logistical challenges to study them in nocturnal contexts^1–5^. Nocturnal behavior in bees has independently evolved at least 19 times in 5 of the 7 known families and in more than 250 species, most of them occurring in the American tropics^6^. Studies indicate that nocturnal bees are responsible for pollinating economically and culturally important plants in the Middle East and Mediterranean regions, such as the caper bush (*Capparis* spp., Capparaceae)^7,8^, as well as cultivated pumpkins and other squash plants in Meso- and North America^9^ and at least 20 other crops in Brazil^4^. Nocturnal bees are also important pollinators of native plants, as in the case of nocturnal sweat bees of the genus *Megalopta* (Smith) (Halictidae), which inhabit lowland tropical forests and visit at least 65 plant species^10^. Thus, nocturnal bees contribute to both ecosystem function and food production.

Several studies have documented changes in bee community composition, population vigor, distribution, and interactions with host plants due to landscape-level alterations and climate change^11,12^. However, the vulnerability of bees to climate change in tropical areas, where the effects are expected to be greater due to organisms living closer to their thermal maxima and low acclimation capacity, are poorly documented^13–15^. Given that global warming models predict that night-time temperatures will increase at a faster rate than day temperatures^16^, the effects of climate change will be greater for tropical nocturnal organisms, especially if they display lower heat tolerance than diurnal taxa. Data on the thermal tolerance of nocturnal insects are scant, but they support the prediction that nocturnal taxa have a lower heat tolerance than diurnal taxa^8,17^. These results are alarming because they suggest that physiological limitations may present an additional challenge for nocturnal ectotherms, some of which are key in vital ecosystem services such as pollination.

We assessed the thermal tolerance of *Megalopta*, a group of nocturnal bees that are restricted to forested areas in the American tropics^18^. We conducted our assays in a lowland Panamanian forest and used dynamic (ramping temperatures) and static (constant temperatures) protocols to assess bees’ thermal tolerance. In the dynamic protocol, we estimated bees’ critical thermal minimum (CT_Min_) and maximum (CT_Max_), the minimum and maximum temperatures at which an animal can maintain muscle control^19,20^. In the static protocol, we measured bee survival after constant heat exposure, which assesses bees’ potential vulnerability to a heat stress event. Such events are predicted to be stronger and more frequent under climate change scenarios^21^. Given that temperature is lower at night than during the day, we predicted that nocturnal bees would display a lower CT_Max_ than diurnal bees. We predicted similar average estimates of CT_Min_ between nocturnal and diurnal bees given that nights in lowland tropical forests are not extremely cold and that the magnitude of the daily variation in temperature is relatively small (see “Results” below) when compared to that experienced in other tropical ecosystems.

Critical thermal limits are strong predictors of an organism's thermal tolerance^15,22,23^, but they are influenced by a myriad of biotic and abiotic factors, including body size, age, nutrition, reproductive status, and temporal and environmental gradients^24,25^. Thus, we also wanted to assess the effect of body size and reproductive status in nocturnal bees. *Megalopta* is a good model organism to explore the effect of these biotic factors because of extensive intraspecific variation in body size, which is related to their flexible social behavior. *Megalopta* nests inside dead branches or lianas found in the forest understory. Females are facultatively social, such that some live as solitary nesters while other live in small social groups of up to 11 females, one of which is typically larger, with well-developed ovaries, and reproductively dominant^18,26–28^. Because small bees cool down and heat up more quickly than large bees due to their high surface area to volume ratio^29,30^, we predicted that CT_Min_ would decrease while CT_Max_ would increase (higher cold and heat tolerance) with increasing body size. Workers and solitary reproductives of *Megalopta* might experience the greatest variance in temperature given that they forage, even though foraging is restricted to short periods during the day, approximately 45 min after sunset and 90 min before sunrise^31^. Unlike workers and solitary reproductives, queens rarely leave the nest^32^. Thus, we predicted that workers and solitary reproductives would display lower CT_Min_ and higher CT_Max_ than queens.

Finally, we used the CT_Max_ derived from our experiments to calculate the warming tolerance (the difference between CT_Max_ and the ambient temperature) of each species and to assess their vulnerability to global warming. The smaller the warming tolerance, the more susceptible an organism is to global warming^13,33^. Although most climate change studies emphasize the role of temperature, desiccation tolerance (the ability of an organism to reduce water loss) may be equally important^34^. For some insects, desiccation stress is the main factor determining their distribution and behavior^35,36^ and climate change is expected to significantly alter precipitation patterns, especially in the tropics^21^. Thus, we were also interested in assessing the desiccation tolerance of nocturnal bees. Given that relative ambient humidity is lower during the day than at night, we predicted that nocturnal bees would be less tolerant to desiccation than diurnal bees.

## Results

### Ambient and nest temperature and humidity

**Figure 1:**
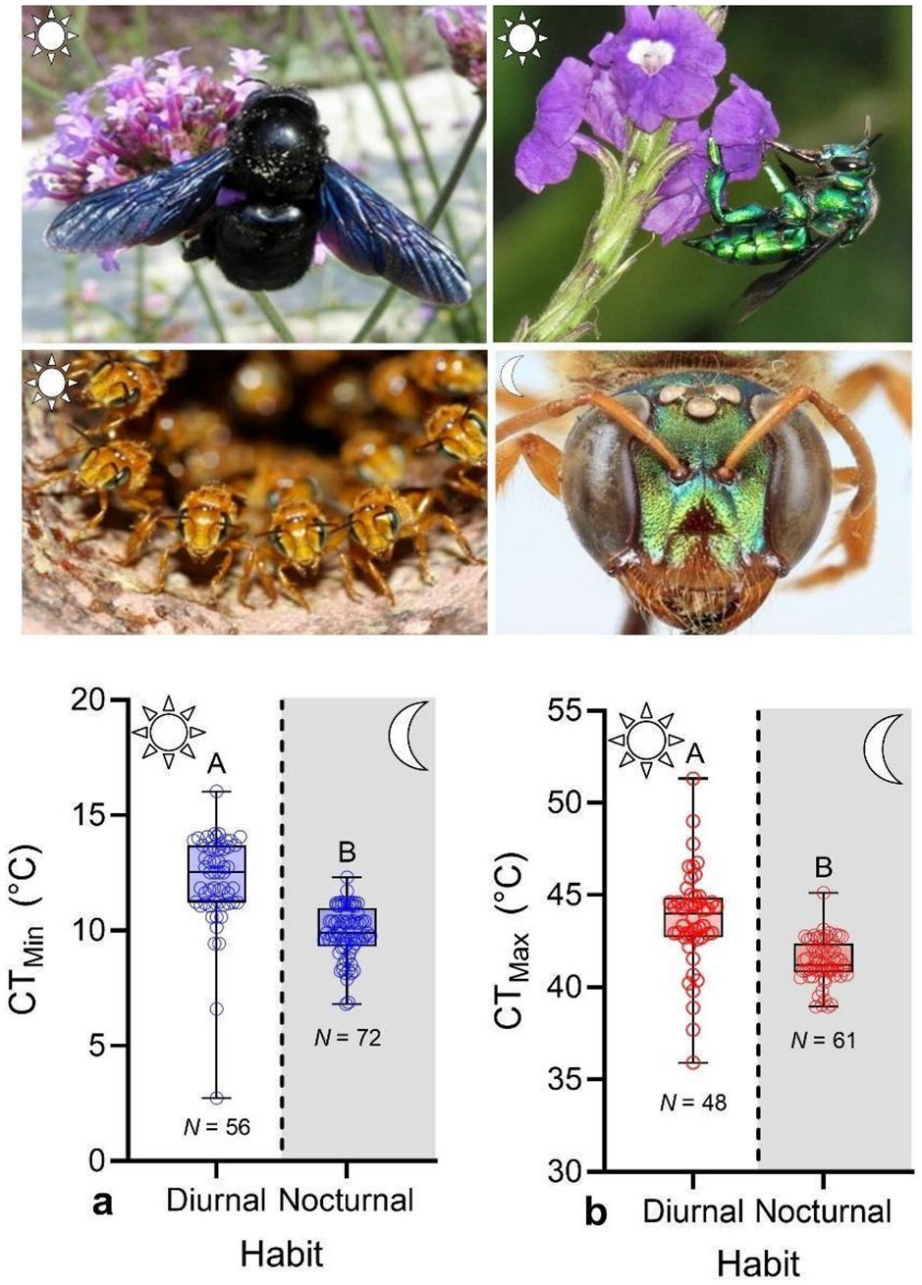
Critical thermal minima (CT_Min_) and maxima (CT_Max_) between diurnal and nocturnal bee species. Box plots show median, quartiles, and extreme values of temperatures. For each thermal limit, a different letter above bars indicates significant differences (*P* < 0.05). Species groups shown, clockwise from upper left, are carpenter bees (Apidae: Xylocopini), orchid bees (Apidae: Euglossini), nocturnal bees (Halictidae: Augochlorini, *Megalopta*), and stingless bees (Apidae: Meliponini).

Temperature and relative humidity differed significantly between day and night periods (Temperature: Wald χ^2^ = 3116.9; Humidity: Wald χ^2^ = 277.6; in both cases *DF* = 1, *P* < 0.001). The mean hourly air temperature during the daytime was 25.3 °C (± 0.03, range: 21.9–29.1, *N* = 3563) whereas that of the night-time was 23.6 °C (± 0.02, range: 21.6–28.1, *N* = 3697; Fig. 1Sa). Mean hourly relative humidity was significantly lower during the day (89.0% ± 0.18, range: 62.0–100.0, *N* = 3563) than at night (92.4% ± 0.13, range: 61.7–100.0, *N* = 3697; Fig. 1Sb).

**Figure 2:**
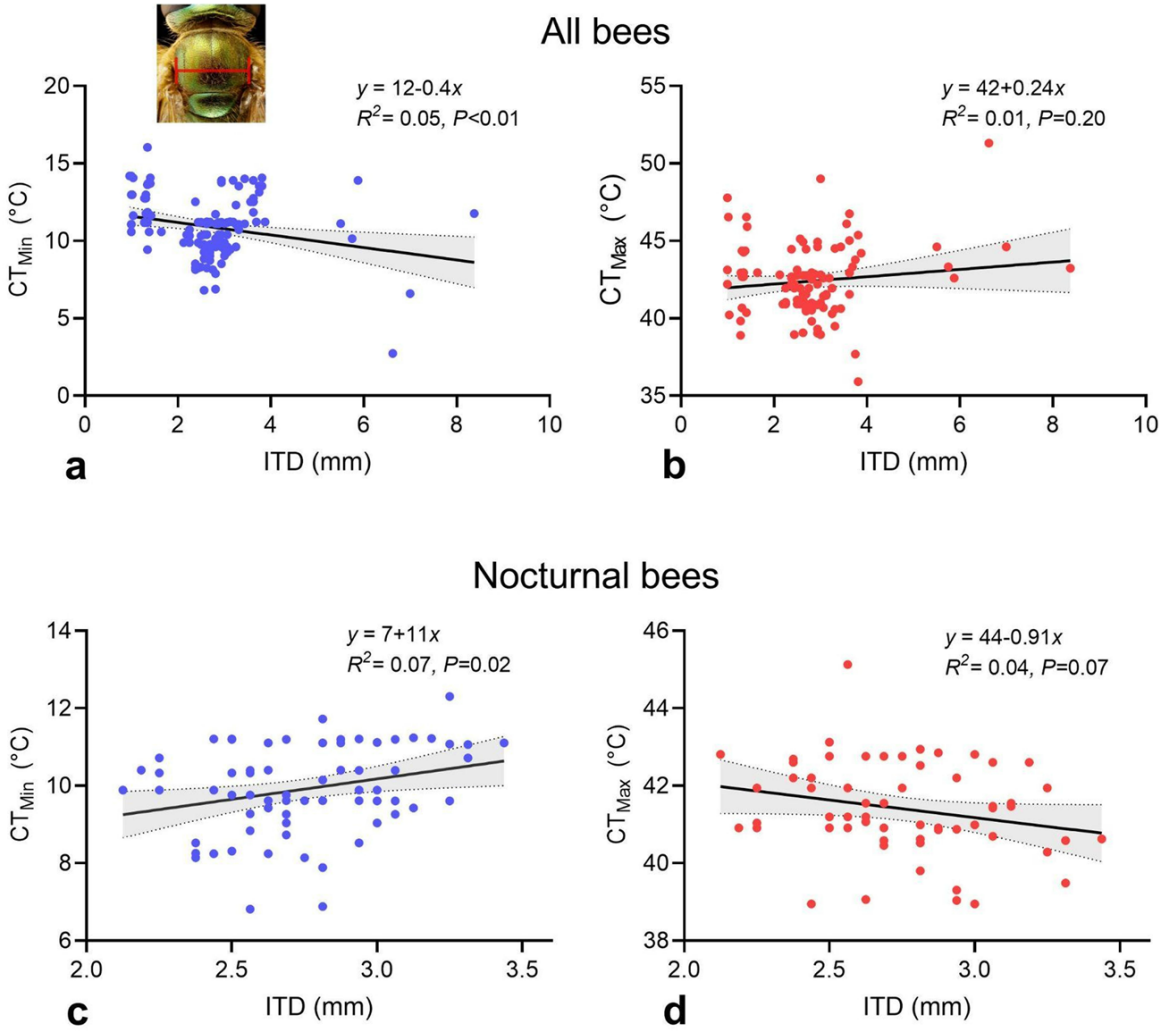
Critical thermal minima (CT_Min_) and maxima (CT_Max_) and their relationship with intertegular distance (ITD) across all bees (**a**, **b**) and in nocturnal bees (**c**, **d**). The trend line represents a linear regression and the grey areas around the line are 95% confidence intervals.

The average internal nest temperature of occupied solitary nests of nocturnal bees was between 0.8 and 1 °C lower than the ambient temperature, and such a difference was significant (χ^2^ = 56.3, *DF* = 3, *P* < 0.001). During the monitoring period, internal nest temperature closely tracked changes in ambient temperature (Fig. 2Sa, b).

### Critical thermal limits and phylogenetic signal

**Figure 3:**
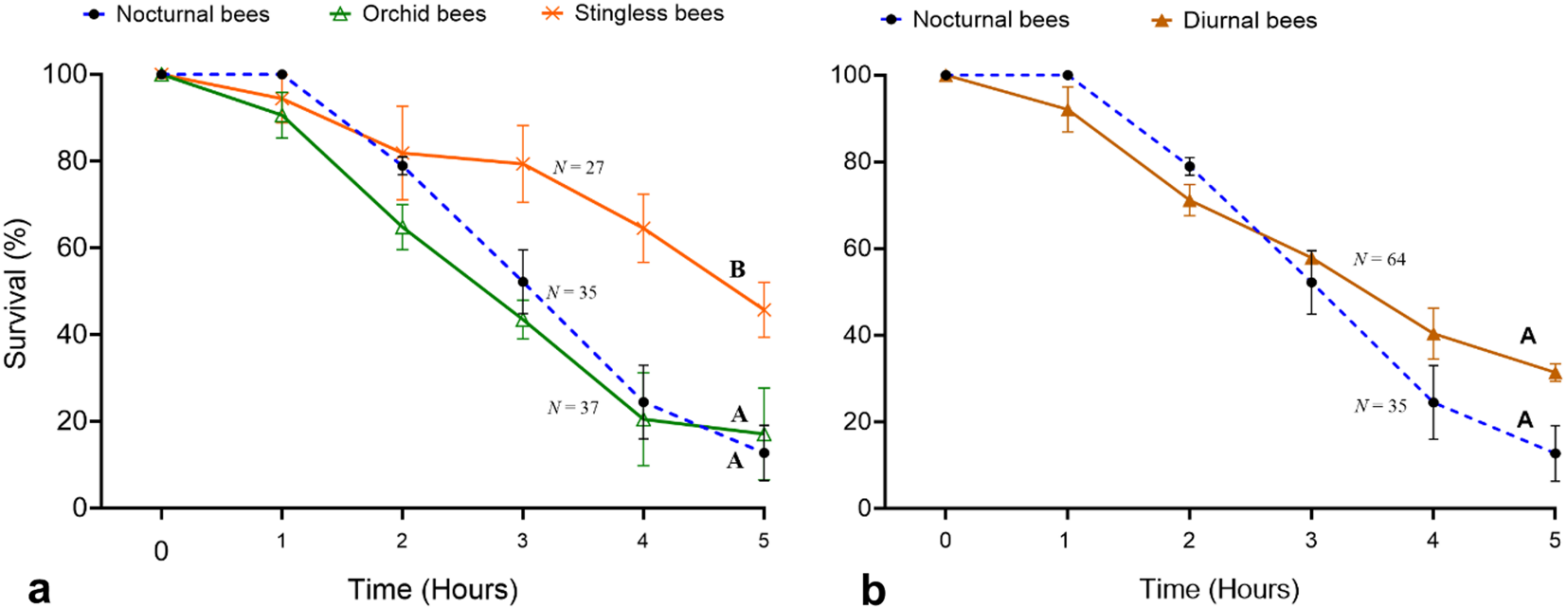
Survival (means ± SE) of nocturnal and diurnal bees after exposure to a heat stress event (36 °C) over 5 h. (**a**) Comparison among groups of bees; (**b**) comparison between nocturnal and diurnal bees (orchid bees and stingless bees pooled together). For each figure, different letters at the end of each survival curve indicate significant (*P* < 0.05) mean differences based on post hoc pairwise comparisons with a Log-rank test.

Nocturnal bees displayed a mean CT_Min_ of 9.92 °C (± 0.132, *N* = 72), which is 2.3 °C lower than the average CT_Min_ of diurnal bees (12.17 ± 0.132, *N* = 56, Fig. 1a). CT_Min_ varied significantly across species (χ^2^ = 144.1, *DF* = 11, *P* < 0.001; Table 1, Fig. 3S) and the difference in CT_Min_ between nocturnal and diurnal bees was significant after accounting for body size (χ^2^ = 152.2, *DF* = 1, *P* < 0.001). Similarly, nocturnal bees displayed a mean CT_Max_ of 41.40 °C (± 0.153, *N* = 61), which is 2.3 °C lower than the average CT_Max_ of diurnal bees (43.37 ± 0.386, *N* = 48, Fig. 1b). CT_Max_ varied significantly across species (χ^2^ = 170.2, *DF* = 15, *P* < 0.001; Table 1, Fig. 3S) and the difference in CT_Max_ between nocturnal and diurnal bees was also significant after accounting for body size (χ^2^ = 143.8, *DF* = 1, *P* < 0.001). *Megalopta* differed from some diurnal species in both CT_Min_ and CT_Max_, but we found no significant differences in these traits between the two species of *Megalopta* (Table 1S). Thermal breadth (TB, difference between CT_Min_ and CT_Max_) differed among species (χ^2^ = 465.6, *DF* = 15, *P* < 0.001, Table 1) but it was similar between diurnal and nocturnal bees after accounting for body size (χ^2^ = 3.43, *DF* = 1, *P* = 0.37, Table 1). CT_Min_, CT_Max_ and TB also differed among broader taxonomic groups (CT_Min_: χ^2^ = 207.5, CT_Max_: χ^2^ = 174.0, TB: χ^2^ = 145.0, *DF* = 3 and *P* < 0.001 in all cases). Nocturnal bees displayed a CT_Min_ similar to that of carpenter bees but significantly lower than that of stingless bees and orchid bees. In contrast, CT_Max_ of nocturnal bees was lower than that of the remaining bee groups (Fig. 3S). Thermal breadth was similar among taxonomic groups, except for that of carpenter bees that was significantly broader. While CT_Min_ displayed significant phylogenetic signal (Pagel’s *λ* = 0.99, *P* = 0.03), CT_Max_ did not (*λ* < 0.01, *P* = 1.0).

**Table 1:** Critical thermal minimum (CT_Min_) and maximum (CT_Max_), sex (Male, Female or Both), thermal breadth (TB), intertegular distance (ITD), and number of nests (Ns) used per species.

Species	Sex	CT_Min_ (°C)	CT_Max_ (°C)	TB (°C)	WT (°C)	ITD (mm)	Ns
Diurnal bees							
*Ceratina currani* Schwarz	F	11.23 ± 0.01, *N* = 2	44.70 ± 0.24, *N* = 2	33.47 ± 0.23, *N* = 2	7.70 ± 0.24, *N* = 2	2.66 ± 0.03, *N* = 2	—
*Eufriesea pulchra* (Smith)	M	12.03 ± 1.88, *N* = 2	42.97 ± 0.36, *N* = 2	30.94 ± 2.24, *N* = 2	5.97 ± 0.36, *N* = 2	5.81 ± 0.06, *N* = 2	—
*Euglossa bursigera* Moure	M	9.43, *N* = 1	49.02, *N* = 1	39.59, *N* = 1	12.02, *N* = 1	3.0, *N* = 1	—
*E. crassipunctata* Moure	M	13.40 ± 0.44, *N* = 3	44.67 ± 0.14, *N* = 3	31.28 ± 0.34, *N* = 3	7.67 ± 0.14, *N* = 3	2.75 ± 0.19, *N* = 3	—
*E. cybelia* Moure	M	13.91, *N* = 1	46.75, *N* = 1	32.84, *N* = 1	9.75, *N* = 1	3.63, *N* = 1	—
*E. imperialis* Cockerell	M	12.53 ± 0.54, *N* = 5	40.98 ± 1.74, *N* = 5	28.45 ± 2.17, *N* = 5	3.98 ± 1.74, *N* = 5	3.78 ± 0.03, *N* = 5	—
*E. mixta* Friese	M	12.29 ± 0.23, *N* = 3	44.69 ± 0.92, *N* = 3	32.40 ± 0.71, *N* = 3	7.69 ± 0.92, *N* = 3	3.60 ± 0.02, *N* = 3	—
*E. tridentata* Moure	M	13.47 ± 0.27, *N* = 6	43.80 ± 0.69, *N* = 5	30.42 ± 0.43, *N* = 5	6.79 ± 0.69, *N* = 5	3.50 ± 0.09, *N* = 6	—
*Eulaema bombiformis* (Packard)	M	9.18 ± 2.59, *N* = 2	43.92 ± 0.69, *N* = 2	34.74 ± 3.28, *N* = 2	6.92 ± 0.69, *N* = 2	7.69 ± 0.69, *N* = 2	—
*Exaerete frontalis* (Guérin-Méneville)	M	11.11, *N* = 1	44.62, *N* = 1	33.51, *N* = 1	7.62, *N* = 1	5.50, *N* = 1	—
*Partamona orizabaensis* (Strand)	F	10.59, *N* = 1	42.95, *N* = 1	32.36, *N* = 1	5.95, *N* = 1	1.64, *N* = 1	—
*Tetragona ziegleri* (Friese)	F	12.30 ± 0.39, *N* = 16	42.38 ± 0.52, *N* = 13	30.44 ± 0.69, *N* = 13	5.38 ± 0.52, *N* = 13	1.33 ± 0.01, *N* = 16	1
*Tetragonisca angustula* (Latreille)	F	12.53 ± 0.49, *N* = 9	43.97 ± 1.40, *N* = 5	32.50 ± 1.42, *N* = 5	6.97 ± 1.40, *N* = 5	1.01 ± 0.01, *N* = 9	1
*Trigona fuscipennis* Friese	F	13.02 ± 1.20, *N* = 3	45.12 ± 1.12, *N* = 3	32.10 ± 1.51, *N* = 3	8.12 ± 1.12, *N* = 3	1.39 ± 0.02, *N* = 3	
*Xylocopa aeneipennis* (De Geer)	F	2.73, *N* = 1	51.32, *N* = 1	48.59, *N* = 1	14.32, *N* = 1	6.63, *N* = 1	1
Nocturnal bees							
*Megalopta amoena* (Spinola)	B	10.00 ± 0.26, *N* = 15	41.85 ± 0.43, *N* = 14	31.83 ± 0.48, *N* = 14	4.85 ± 0.43, *N* = 14	2.41 ± 0.04, *N* = 15	10
*M. genalis* Meade-Waldo	B	9.90 ± 0.15, *N* = 57	41.26 ± 0.15, *N* = 47	31.26 ± 0.24, *N* = 47	4.20 ± 0.15, *N* = 47	2.85 ± 0.03, *N* = 57	44

— = non-applicable, as bees were collected with odor baits or from flowers. For each trait, mean value is followed by SE and number of individuals measured.

### Critical thermal limits and morphological traits

Body size, measured as ITD, varied among species, from 1.01 mm in the stingless bee *Tetragonisca angustula* to 7.69 mm in the large carpenter bee *Eulaema bombiformis* (Table 1). CT_Min_ decreased with increasing ITD across all species (Fig. 2a; *P* < 0.01, *R*^2^ = 0.05) and in diurnal bees alone (*P* = 0.01, *R*^2^ = 0.10). In contrast, CT_Min_ increased with increasing ITD in nocturnal bees (Fig. 2c; *P* = 0.02, *R*^2^ = 0.07). CT_Max_ did not increase significantly with ITD across all species (Fig. 2b; *P* = 0.20, *R*^2^ = 0.01), nocturnal bees (Fig. 2d; *P* = 0.07, *R*^2^ = 0.04), or diurnal bees (*P* = 0.24, *R*^2^ = 0.01).

### Critical thermal limits and caste comparison in *Megalopta*

**Figure 4:**
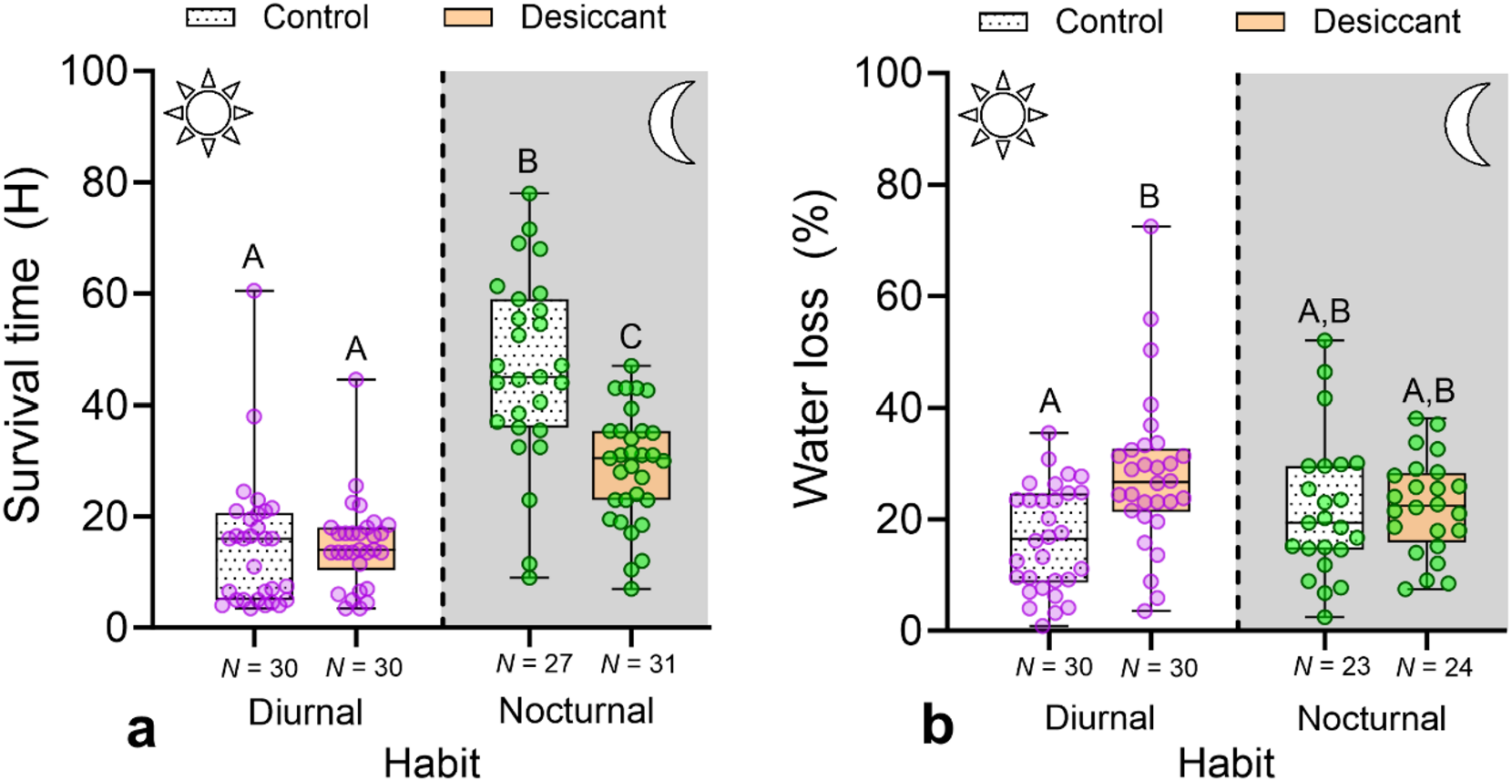
Survival time (**a**) and percentage of water loss (**b**) between nocturnal and diurnal bees exposed to a desiccant (colored boxes) or control (white, stippled boxes). Box plots show median, quartiles, and extreme values. For each figure, a different letter above bars indicates significant differences (*P* < 0.05).

Head width and ovary area, morphological features related to social and reproductive status in *Megalopta*, were significantly correlated (*r* = 0.56, *N* = 70*, P* < 0.001). While CT_Min_ increased significantly with increasing head width (Fig. 4Sa; P = 0.01, *R*^2^ = 0.07) and ovary area (Fig. 4Sc; *P* < 0.01, *R*^2^ = 0.10), CT_Max_ did not (Head width: *P* = 0.10, *R*^2^ = 0.01; ovary area: *P* = 0.49, *R*^2^ = − 0.01; Fig. 4Sb, d). After accounting for body size, CT_Min_ differed among solitary reproductives, queens, and workers (χ^2^ = 3.58, *DF* = 2, *P* = 0.03; Table 2S, Fig. 5Sa). Pairwise comparisons indicated differences only between queen and worker, as the latter displayed an average estimate of CT_Min_ of 9.53 °C (± 0.26, *N* = 17), which is about 1.2 °C lower than the average estimate for the queen 10.69 °C (± 0.22, *N* = 13). After accounting for body size, CT_Max_ was similar among solitary reproductives, queens, and workers (χ^2^ = 0.48, *DF* = 2, *P* = 0.62; Fig. 5Sb).

### Bee survival under acute heat exposure

Bee survival under acute heat exposure differed among stingless bees, orchid bees, and nocturnal bees (χ^2^ = 10.4, *DF* = 2, *P* < 0.01). Pairwise comparisons indicated that stingless bees displayed greater survival than orchid bees and nocturnal bees, which did not differ from each other (Fig. 3a, Tables 3S, 4S). According to the hazard ratio (HR: 0.41), mortality in stingless bees was 59% lower than in nocturnal bees. Median survival time was 3, 4, and 5 h for orchid bees, nocturnal bees, and stingless bees, respectively. However, when data from diurnal bees are analyzed together, bee survival is similar between diurnal and nocturnal bees (χ^2^ = 1.6, *DF* = 1, *P* = 0.2; Fig. 3b).

### Warming tolerance

Warming tolerance (WT) varied significantly across species (χ^2^ = 169.6, *DF* = 15, *P* < 0.001, Table 1), from 3.98 °C in *E. imperialis* to 14.32 °C in *X. aeneipennis*. Nocturnal bees displayed an average WT of 4.56 °C, which is 3.23 °C lower than that of diurnal bees (7.79 °C). After accounting for body size, such a difference was significant (χ^2^ = 143.8, *DF* = 1, *P* < 0.001).

### Desiccation tolerance

The survival time of bees exposed to a desiccant significantly increased with increasing ITD across all species (Fig. 6S; *P* < 0.001, *R*^2^ = 0.20). However, the relationship between desiccation survival time and ITD was not significant for nocturnal bees alone (*P* = 0.71, *R*^2^ = − 0.03). Bee survival time varied significantly between treatments, habit, and the interaction between treatment and habit, after accounting for body size (Treatment: χ^2^ = 15.3, Habit: χ^2^ = 67.9, Treatment × Habit: χ^2^ = 17.3; *P* < 0.001, *DF* = 1 in all cases; Table 5S). Pairwise comparisons indicated that nocturnal bees exposed to a desiccant survived about half as long as control bees (28.98 h ± 1.83, *N* = 31 vs 46.45 h ± 3.26, *N* = 27), and almost two times longer than diurnal bees exposed to either treatment (Fig. 4a; Tables 5S, 6S). For diurnal bees, the survival time was similar between the control and treatment (~ 15 h).

The percentage of water loss was similar between diurnal and nocturnal bees but varied significantly between treatments and the interaction between these two factors (Treatment: χ^2^ = 9.00, *P* = 0.003; Habit: χ^2^ = 0.01, *P* = 0.94; Treatment × Habit: χ^2^ = 6.09, *P* = 0.02, *DF* = 1 in all cases; Table 4S). Pairwise comparisons indicated differences only between the control and treatment of diurnal bees (Fig. 4b; Table 6S). On average, water loss was 12% higher in diurnal bees exposed to the desiccant than the control.

## Discussion

Our results demonstrate that nocturnal bees of the genus *Megalopta* display, on average, lower CT_Min_, CT_Max_, and warming tolerances than the species of diurnal bees we assessed (Fig. 1, Table 1). However, survival in both nocturnal and diurnal bees was significantly reduced following acute heat exposure using realistic temperatures (36 °C) that bees might experience in their natural habitats (Fig. 3). Thus, these results partially agree with our predictions that nocturnal bees are generally more thermally sensitive than diurnal bees. The decrease in both CT_Min_ and CT_Max_ displayed by nocturnal bees was on average 2.3 °C, which is the difference between the day and night mean hourly temperature we recorded in the forest understory. However, a decrease in CT_Max_ as small as 1 °C has been recorded in the nocturnal *X. olivieri* relative to two other diurnal carpenter bee species^8^, whereas a decrease as large as 4 °C has been documented in nocturnal ants in Mexico relative to diurnal species^17^. Thus, our results are within the magnitude of change in CT_Max_ displayed by other nocturnal insects. Similarly, the warming tolerances recorded for bees in our study are within the range of other tropical insects^13^.

The low CT_Max_ and small warming tolerances displayed by nocturnal bees not only suggest high vulnerability to gradual global warming, but also to changes in microclimate due to anthropogenic factors. For example, changes in forest coverage due to selective logging can significantly increase temperature in the forest understory^37^, thus potentially creating unsuitable habitats for *Megalopta*. The internal nest temperature of *Megalopta* is 0.8–1.0 °C lower than the ambient temperature, and it closely tracks variations in ambient temperature (Fig. 2S). Thus, nests provide little buffer from ambient temperatures, and because adults remain inside the nest most of the day, nest selection might be an important factor for these bees. This could explain why nests of *Megalopta* are usually found in well-shaded areas within the forest understory and rarely at other strata or along forest edges or clearings. This is different from nests of other twig-nesting bees (e.g., *Ceratina*, *Xylocopa*) that are common in open, sun-exposed areas and whose adults display a higher CT_Max_^38,39^.

The results from our acute heat exposure assay suggest that both diurnal and nocturnal bees might be similarly impacted by transient warming events and heatwaves in lowland tropical forests, but particularly *Megalopta* and orchid bees (Fig. 3). Stingless bees appear to be less susceptible despite their small body size. In addition, stingless bees can thermoregulate their nests^40,41^, which might provide them with greater plasticity to tolerate or to adapt to changes in climate when compared to solitary bees. It would be interesting to assess whether social nests of *Megalopta* display some type of active thermoregulatory behavior, such as fanning, typical of social bees^40,42^. In pilot assays, pairs of bees inside circle tube arenas under an acute heat exposure displayed trophallaxis (food exchange among adults), which has never been observed under the same experimental set up at ambient temperatures (WTW, VHG pers. obs.), although the behavior is known to occur in observation nests at ambient temperatures^32^. This observation suggests that trophallaxis might be a mechanism used by *Megalopta* to increase their evaporative cooling capacity and mitigate heat stress.

CT_Min_ is not measured as frequently as CT_Max_ in bee thermal studies^43^, and thus we do not know if other nocturnal bees also show lower CT_Min_. However, the ability of nocturnal bees to fly at low temperatures has already been noted in the literature, thus suggesting that this might be the case^44,45^. Given that thermal breadth was similar between nocturnal and diurnal bees, a decrease in CT_Max_ was likely associated with a decrease in CT_Min_. A decrease in both thermal traits could have been the result of less selection for high-temperature stress accompanied by an increase in selection for performance at relatively low temperatures. Although temperatures at the study site^46^ never approach *Megalopta*’s CT_Min_ and CT_Max_, negative effects of temperature on bees’ performance and behavior can be seen well before they reach their thermal limits^47^. Thus, while the environment at the study site does not approach the CT_Min_ of any of the species in our study, nocturnal bees may have better flight performance at relatively cooler temperatures than some diurnal species with higher CT_Min_, if such temperatures occur when there are enough photons (light levels) for their visual systems to function. *Megalopta* cannot forager later in the evening or earlier in the morning to take advantage of cooler temperatures because there is not enough light then for them to navigate^31^.

CT_Min_ displayed a strong phylogenetic signal, suggesting that closely related species in our study exhibited more similar CT_Min_ than distantly related species (Figs. 7S, 8S). This is the case for orchid bees and stingless bees, which contained most of the species assessed in the study. In contrast, no phylogenetic signal was detected for CT_Max_. Future studies should assess if the low CT_Min_ observed in *Megalopta* is also displayed by other nocturnal bees, as well as if this thermal trait is phylogenetically restricted to particular clades within the bee family Halictidae. For example, *M. atra* Engel, the sister group of the rest of *Megalopta*, is a species restricted to montane habitats (1160–1235 m) in Costa Rica and Panama^48^, suggesting that cold tolerance might be a conserved trait in this clade. Unfortunately, we were not able to assess the thermal limits of other sweat bee species and CT_Min_ data are not available for any other sweat bee species^43^.

We found that CT_Max_ did not increase with increasing body size, head width, or ovary area (Figs. 2b, d,  4Sb, d); it was also similar among queens, workers, and solitary reproductive (Fig. 5S). In contrast, CT_Min_ decreased with increasing body size across all bees (Fig. 2a) but it increased with increasing body size (Fig. 2c), head width (Fig. 4Sa), and ovary area (Fig. 4Sc) in *Megalopta*. Queens displayed a CT_Min_ 1.2 °C higher than workers, but similar to solitary reproductives (Fig. 5S). Therefore, these results are partially in agreement with our predictions that CT_Min_ decreases while CT_Max_ increases with increasing body size, and that both workers and solitary reproductives would display a lower CT_Min_ and a higher CT_Max_ than queens. In some species of bumble bees, CT_Min_ decreases and CT_Max_ increases with increasing body size^30^, while in other bees there is no effect of body size on heat tolerance^8,30,38,43^. In stingless bees, CT_Max_ increases with increasing body size^41^ whereas in a Mediterranean bee community, CT_Max_ does not increase with increasing body size (VHG., unpublished results), as documented here across all bees. Thus, the relationship between thermal limits and body size in bees appears to be complex, as they vary among species, clades, and communities^41,43,49^.

Although the increase in CT_Min_ with increasing body size in *Megalopta* was unanticipated, CT_Min_ also increases with increasing body size across species of neotropical stingless bees^41^, as well as in some species of fruit flies, at least at the population level in the latter taxon^50^. Thus, the relationship between CT_Min_ and body size reported here for *Megalopta* is not uncommon among insects. Our data also suggest that reproductive status might influence cold tolerance in *Megalopta*, as CT_Min_ increased with increasing ovary area, and queens displayed an average CT_Min_ higher than workers, but similar to solitary reproductives (Fig. 5Sa). These results agree with studies on the Asian lady beetle (*Harmonia axyridis*, Coccinellidae) demonstrating that unmated individuals and mated individuals that had not reproduced displayed greater cold tolerance than mated and reproducing individuals^51^. Thus, it is possible that reproduction induces physiological changes in females that reduce their cold tolerance, although we cannot rule out entirely that such differences between workers, queens, and solitary reproductives might be also due to the thermal environment they experience. Workers and solitary reproductives are likely exposed to the greatest variance in temperature given their role in foraging when compared with queens, which rarely leave the nest^32^. Because thermal limits predict foraging temperatures^52^, workers might be able to forage at cooler temperatures than queens. These observations are relevant to understand other aspects of the social biology of *Megalopta* and are aligned with the idea of within-group variation in the thermal capacities across colony members in social insects, as it has been documented in ants^53,54^.

The results from the desiccation tolerance assays are puzzling. The survival time between the control and treatment for diurnal bees was similar, while that of nocturnal bees decreased by half in the treatment group (Fig. 4a). Taken independently, these results could be interpreted as indicating low desiccation resistance in *Megalopta*, aligning with our initial prediction. However, when comparing the survival time between diurnal and nocturnal bees, the latter survived almost twice as long after desiccant exposure, even when controlling for body size. Therefore, in comparison to diurnal bees, we interpret that *Megalopta* displays greater desiccation resistance. Although unanticipated, our results agree with observations indicating higher desiccation tolerance in the sweat bee *Agapostemon sericeus* (Foster) when compared to bumble bees and honey bees in North America^55^. A similarly high desiccation resistance has been observed in the European sweat bee *Lasioglossum malachurum* (Kirby) when compared to honey bees in Greece (VHG pers. obs). Thus, desiccation resistance might be a phylogenetically conserved trait, although measurements of more species of sweat bees in different environments are required to test this hypothesis. Our results also suggest a trade-off between thermal tolerance and desiccation tolerance, in which species with high CT_Max_, such as stingless bees, will display low desiccation tolerance. This pattern has been observed in tropical canopy ants from the same site where we conducted our studies^36^ and might be related to having a permeable cuticle that allows ants to engage in passive evaporative cooling when temperature increases. Similar responses have been documented among three species of bees in North America, thus supporting this trade-off between thermal tolerance and desiccation tolerance^55^. Future studies should explore this aspect as well as focus on understanding the potential effects of desiccation on bees’ thermal tolerance. At least in some insects, desiccation reduces heat tolerance^56,57^, and thus it might increase the vulnerability of nocturnal bees to climate change.

It is important to note that we conducted our study from a single population of bees and in a narrow temporal window, and thermal limits are known to vary spatially and temporally^24,25^. In addition, we used a reduced number of diurnal and nocturnal species, some of which were represented by only one of the sexes and a single individual in our experiments. Thus, future studies should address these issues at greater spatial and temporal scales, although at our site, as with many in the lowland tropics, temperature varies little throughout the year. Despite these limitations, our results are consistent with other studies and shed light on the potential impact of global warming on nocturnal tropical insects. For example, plant reproductive biology (e.g., pollen tube growth) is highly sensitive to temperature^58^, and therefore temperature-dependent synchronization with pollinators is critical. Consequently, our results have significant implications for our current understanding of the potential effects of climate change on nocturnal pollinators and their pollination services. We showed that nocturnal sweat bees have lower CT_Max_ than diurnal bees. Because our results agree with previous works on nocturnal ants and unrelated bee taxa, such as carpenter bees, it is likely that low heat tolerance might be a widespread phenomenon among other nocturnal insect pollinators, such as moths, flies, and beetles. This is ecologically and economically concerning because in some cases nocturnal pollinators offer a comparable contribution to that of diurnal pollinators^3,5,59^ and, even in cases where they provide redundancy to diurnally pollinated plants^2^, the loss of a single pollinator may disrupt an entire network^60^. Future studies should assess the acclimation capacity of nocturnal pollinators, as such plastic responses can potentially compensate for the negative consequences of climate change^34^.

Tropical insects are expected to display limited acclimation capacity^13–15^ and a recent study supports this idea^61^. However, nocturnal pollinators might display greater acclimation capacity relative to diurnal species considering their low CT_Max_ and the limited opportunities for behavioral thermoregulation, which might favor the evolution of greater physiological plasticity^62^. Although the thermal tolerance of pollinators is still poorly known^8,43^, even less information is available on their desiccation tolerance. Thus, future studies should not only assess this physiological trait, but also should assess how desiccation and other stressors (e.g., nutrition, pesticides, etc.) might influence insect thermal tolerance. As droughts continue to increase in frequency and duration, drier conditions might make insects less thermally tolerant, as has been documented in fruit flies^56^ and ants^57^. Finally, future studies should also assess the behavioral responses of nocturnal insects to climate change. Social behavior is expected to provide insects with a greater behavioral plasticity to tolerate environmental changes^63,64^, and the facultative social behavior of *Megalopta* provides a unique opportunity to explore this idea.

## Materials and methods

### Study site and bee collections

We conducted field and experimental work during the beginning of the dry season (January–February 2022) on Barro Colorado Island (BCI), Republic of Panama (9°9′ N, 79°51′ W). We used *Megalopta amoena* and *M. genalis*, the two most common nocturnal bees at the study area. Nests were collected during the day when adults were inside. We plugged nest entrances with cotton balls and transported them to the laboratory, where we opened them with a pocketknife and extracted adult bees. We collected stingless bees (Apidae: Meliponini), orchid bees (Apidae: Euglossini), and carpenter bees (Apidae: Xylocopinini) as representatives of diurnal bees. We chose these bees because they were common or easy to capture on our study site. Male orchid bees were attracted with odor baits, and all other bees, which are females, were collected either when entering their nests or at flowers with the aid of an insect net. We then transferred bees individually to a plastic vial, which we then capped with fabric (~ 1 mm mesh). We kept bees inside a Styrofoam cooler with an ice pack covered in a piece of cloth (16–19 °C) until we completed fieldwork. We tested bees within 1–2 h after being captured in the field. In all assays, we used the two common species of nocturnal bees, but because bees were collected opportunistically, the number and identity of diurnal bees differed among experiments depending on their availability in the field.

### Ambient and nest temperature and humidity

To characterize the microclimate where nocturnal bees nest, we measured ambient temperature and relative humidity using iButton data loggers (DS1923 Hygrochron™; Maxim Integrated, San Jose, California) at about 1 m above ground in the forest understory. We set up five data loggers five meters apart, each protected from solar radiation with aluminum foil, and hung from tree branches (Fig. 9S). We recorded temperature and humidity every 5 min for five consecutive days (10–15 January 2022).

To characterize changes in the internal nest temperature of *Megalopta*, we monitored the temperature of three occupied solitary nests every 5 min during four consecutive hours, from 9:30 to 13:30 h. We placed a K-type thermocouple inside the tunnel of each nest and individually tracked them using a TC-08 thermocouple data logger (Pico Technology, Tyler, TX, USA). We took these measurements during the day, inside the shaded forest understory, and placed a thermocouple next to the observation nests to simultaneously record ambient temperature.

### Critical thermal limits assays

We measured bees’ heat and cold tolerances using the Elara 2.0 (IoTherm, Laramie, WY, https://www.iotherm.net/), a fully programmable heating/cooling anodized aluminum stage designed for precision temperature control of laboratory and field samples. We placed bees individually inside glass vials (either 9 × 30 mm, 0.92 cm^3^ for small bees or 12 × 35 mm, 1.85 cm^3^ for larger bees) and plugged each with a moistened cotton ball (~ 0.2 mL of distilled water per cotton ball) to ensure consistent humidity during the assays (Fig. 9S). We used an initial temperature of 22 °C and held bees for 10 min at this temperature before increasing it or decreasing it at a rate of 0.5 °C/min. We chose this rate of temperature change to reduce the time required for each assay and to minimize the effect of confounding physiological stressors, such as dehydration or starvation^43^. We placed vials horizontally on the stage to prevent bees from climbing the sides of the vial. To estimate the temperature inside the vials, we placed a K-type thermocouple inside two empty glass vials plugged with a cotton ball. We individually tracked these vial temperatures using a TC-08 thermocouple data logger. As an approximation of bees’ thermal limits, we used the temperature at which bees show signs of curling (CT_Min_)^20^ or lost muscular control, spontaneously flipping over onto their dorsa and spasming (CT_Max_)^19,65^. Then, after these bioassays concluded, we euthanized specimens to measure morphological and reproductive traits as indicated below. Pilot assays indicated that bees held in similar glass vials, plugged with a moistened cotton ball and adjacent to the Elara 2.0 at room temperature, survived through the duration of the essays. For this experiment, we tested 17 species of 11 genera (Table 1).

### Acute heat event

To assess for differences between nocturnal and diurnal bees’ ability to tolerate an acute heat event, we exposed them to 36 °C and 70% relative humidity inside an incubator (Percival Scientific, Inc., Perry, IA. Model I30VLC8). We chose this temperature because it is about 1 °C lower than the highest average monthly maximum temperature recorded on BCI along a 40 m transect through the forest canopy^46^. We placed bees individually inside plastic vials capped with fabric (1 mm mesh) and monitored their survival every hour for five hours. The response variable in this experiment was time to death. We tested 13 species of 7 genera (Table 7S).

### Warming tolerance

To assess species vulnerability to global warming, we calculated the warming tolerance (WT) as the difference between the CT_Max_ derived from our experiments and the maximum monthly mean (37 °C) recorded on BCI^46^. The smaller WT values, the more susceptible an organism is to global warming^13,33^.

### Desiccation tolerance

To assess for differences between nocturnal and diurnal bees’ ability to tolerate desiccation stress, following an apparatus developed for ants^36^, we placed bees individually in glass vials of 7.4 ml (17 × 60 mm) sealed with a fabric (1 mm mesh) and connected to a vial filled with fully dehydrated Drierite desiccant (W.A. Hammond Drierite Co. Ltd., Xenia, OH). We drilled a 0.5-cm opening on the vial lids, which we glued together with the mesh in between using super glue. We used duct tape to reinforce both vial lids externally and sealed them with parafilm tape. We conducted assays at room temperature (~ 22 °C), monitoring bee survival every hour and recording the time of death as a response variable. As a control, we placed a bee in a similar apparatus, but with the second vial containing a piece of moistened paper towel rather than desiccant (Fig. 9S). We recorded the intertegular distance (ITD) for each specimen. To estimate the percentage of water loss during the assay, we measured bees’ weight before and after the experiment. For this experiment, we tested 8 species of 6 genera (Table 7S).

### Morphological and reproductive traits

As a proxy of body size, we measured the minimum intertegular distance^66^ (ITD) of each specimen used in the thermal limit and desiccation stress assays. In addition, we also measured the maximum head width (HW) of females of *Megalopta* because they display cephalic allometry (i.e., large head and a small thorax), which might play a role in their social biology^26,67,68^. As a proxy of reproductive status, we measured the area of each ovary and used average value in the analyses. As in previous studies^69^, we dissected the abdomen by removing the terga and measured the area of each ovary from digital photomicrographs (10 × magnification) using ImageJ^70^, version 1.51q. We conducted measurements and dissections of the abdomen using an ocular micrometer on an S6E stereomicroscope (Leica Microsystems, Wetzlar, Germany). We categorized females from each social nest (11 of 54 nests) into either queen or worker based on their average ovary area. A bee with the largest ovary area was categorized as a queen while the remaining bees as workers. Females from solitary nests were regarded as solitary reproductives. We measured head width and ovary area of females of *Megalopta* that we used in the thermal limit assays because we were interested in assessing the influence of these morphological traits on bees’ thermal tolerance. We did not assess ovary area for bees used in the acute heat exposure or desiccation stress assay. Voucher specimens are in the insect collection of the Smithsonian Tropical Research Institute, Balboa, Panama.

### Statistical analysis

We conducted statistical analyses in R^71^. To test for differences in air temperatures and relative humidity between daytime and night-time periods (defined, respectively, as the time between sunrise and sunset, and between sunset and sunrise) we implemented a linear mixed-effect model (LMM) using the lmer function in the lme4 package^72^. We used either temperature or humidity as a response variable, period (day or night) as a fixed factor, and datalogger identity as a random factor. To test for differences between ambient and internal nest temperature, we used a one-way ANOVA model with the lm function. To assess for differences in CT_Min_, CT_Max_, thermal breadth (TB), and warming tolerance (WT) between diurnal and nocturnal bees, we used an ANCOVA test by implementing a linear model using the lm function. In this model, we used either CT_Min_, CT_Max_, TB or WT as the response variable, habit (diurnal vs nocturnal) and species as fixed factors, and ITD as covariate. To assess for differences in CT_Min_, CT_Max_ or TB among broader taxonomic groups (nocturnal bees, stingless bees, orchid bees, and carpenter bees), we implemented an ANCOVA test using CT_Min_, CT_Max_ or TB as the response variable, group as fixed factor, and ITD as covariate. To evaluate the relationship between each morphological and reproductive trait (ITD, HW, and ovary area) and CT_Min_ and CT_Max_, as well as between bees’ survival time and body size, we implemented a linear regression analysis using the lm function. We used an ANCOVA test to assess for differences in CT_Min_ and CT_Max_ among solitary reproductives, queens and workers. We used either CT_Min_ or CT_Max_ as the response variable, social status as a fixed factor, and ITD as covariate. To test for differences in the survival time between diurnal and nocturnal bees, we used an ANCOVA test with habit and treatment (control vs desiccant) as fixed factors and ITD as covariate. We implemented a linear model to assess the percentage of water loss between diurnal and nocturnal bees and used habit and treatment as fixed factors. We assessed the significance of fixed effects using a Type II Wald χ^2^ test with the car package^73^. When factors and factor interactions were significant, we used the lsmeans package^74^ to conduct multiple pairwise comparisons with Bonferroni adjustments to assess for differences among groups. We used failure-time analyses to assess differences in bee survival in the acute heat exposure assays. We implemented a Cox proportional hazard model using the survival package^75^, including either taxonomic group or habit as a fixed factor, and conducting post hoc pairwise comparisons with a Log-rank test. To check for the proportional hazard assumption of each Cox model, we tested for independence between time and the corresponding set of scaled Schoenfeld residuals of each variable (treatment and colony identity) using the functions cox.zph in the survival package and ggcoxzph in the survminer package^76^.

### Phylogenetic signal

To account for potential species relatedness effects on critical thermal limits, we build a phylogeny for the focal species using five nuclear and mitochondrial gene fragments (see electronic supplementary methods, Figs. 7S, 8S, Table 8S). We calculate the phylogenetic signal of CT_Min_ and CT_Max_ using Pagel’s λ^77^ with the phylosig function of phytools package^78^. We used 10,000 simulations and a likelihood ratio test to assess for significant departure from 0 (no phylogenetic signal).

### Supplementary Information

Supplementary Information.

## Data Availability

All relevant data are within the paper and its supporting information files. The complete datasets used for the analyses in this study are available on Dryad: 10.5061/dryad.8pk0p2nrz.
