# Supplementary material for: Low heat tolerance and high desiccation resistance in nocturnal bees and the implications for nocturnal pollination under climate change
Source: Sci Rep. 2023 Dec 15;13:22320. doi: 10.1038/s41598-023-49815-6 (PMC10724170; doi:10.1038/s41598-023-49815-6)
Supplement: Supplementary file 1 — Supplementary Information. [file 41598_2023_49815_MOESM1_ESM.pdf]

Supplementary Files

Low heat tolerance and high desiccation resistance in nocturnal bees and the implications for nocturnal pollination under climate change

Victor H. Gonzalez[1*], Rachel Manweiler[1], Adam R. Smith[2], Kennan Oyen[3], David Cardona[4], and William T. Wcislo[4]

[1]Department of Ecology and Evolutionary Biology, University of Kansas, Lawrence, Kansas, 66045, U.S.A. https://orcid.org/0000-0002-4146-1634
[2]Department of Biological Sciences, George Washington University, Washington, U.S.A. https://orcid.org/0000-0002-4291-6928
[3]Animal Diseases Research Unit, Agricultural Research Service, United States Department of Agriculture, Pullman, Washington, 99164, U.S.A. https://orcid.org/0000-0002-5170-6500
[4]Smithsonian Tropical Research Institute, Panama, Republic of Panama

*Author for correspondence (vhgonza@ku.edu)

Running title: Thermal and desiccation tolerance of nocturnal bees

Table 1S. Results of pairwise comparisons with Bonferroni adjustment of the critical thermal minima (CT_{Min}) and maxima (CT_{Max}) among focal species. Only significant ($P < 0.05$) comparisons of the 136 total tests for each thermal trait are listed.

**Comparison**	**CT_{Min} *P*-value**	**Comparison**	**CT_{Max} *P*-value**
M. amoena vs *E. bombiformis*	0.009	*M. amoena* vs *E. bursigera*	0.010
M. amonea vs *T. ziegleri*	<0.0001	*M. genalis* vs *E. bursigera*	0.001
M. amonea vs *T. angustula*	<0.0001	*E. pulchra* vs *X. aeneipennis*	0.013
M. amonea vs *T. fuscipennis*	<0.0001	*E. bursigera* vs *E. imperialis*	0.016
M. amonea vs *X. aeneipennis*	<0.0001	*E. imperialis* vs *X. aeneipennis*	0.014
M. genalis vs *E. crassipunctata*	0.0001		
M. genalis vs *E. bombiformis*	0.01		
M. genalis vs *T. ziegleri*	<0.0001		
M. genalis vs *T. angustula*	<0.0001		
M. genalis vs *T. fuscipennis*	<0.0001		
M. genalis vs *X. aeneipennis*	<0.0001		
C. currani vs *E. bombiformis*	0.003		
C. currani vs *T. angustula*	0.036		
C. currani vs *X. aeneipennis*	< 0.0001		
E. pulchra vs *C. crassipunctata*	0.012		
E. pulchra vs *E. tridentata*	0.011		
E. pulchra vs *E. bombiformis*	0.003		
E. pulchra vs *T. ziegleri*	0.023		
E. pulchra vs *T. angustula*	0.016		
E. pulchra vs *T. fuscipennis*	0.012		
E. pulchra vs *X. aeneipennis*	<0.0001		
E. bursigera vs *T. ziegleri*	0.006		
E. bursigera vs *T. angustula*	0.002		
E. bursigera vs *T. fuscipennis*	0.004		
E. bursigera vs *X. aeneipennis*	<0.0001		
E. crassipunctata vs *E. bombiformis*	0.0001		
E. crassipunctata vs *E. frontalis*	0.012		
E. crassipunctata vs *X. aeneipennis*	<0.0001		
E. cybelia vs *E. bombiformis*	0.0001		
E. cybelia vs *X. aeneipennis*	<0.0001		
E. imperialis vs E. bombiformis	0.0001		
E. imperialis vs *X. aeneipennis*	<0.0001		
E. mixta vs *E. bombiformis*	0.0004		
E. imperialis vs *X. aeneipennis*	<0.0001		
E. tridentata vs *E. bombiformis*	<0.0001		
E. tridentata vs *E. frontalis*	0.017		
E. tridentata vs *X. aeneipennis*	<0.0001		
E. bombiformis vs *P. orizabaensis*	0.011		
E. bombiformis vs *T. ziegleri*	0.0005		
E. bombiformis vs *T. angustula*	0.0005		
E. bombiformis vs *T. fuscipennis*	0.0003		

E. frontalis vs *T ziegleri*	0.015
E. frontalis vs *T. angustula*	0.01
E. frontalis vs *T. fuscipennis*	0.008
E. frontalis vs *X. aeneipennis*	<0.0001
P. orizabaensis vs *X. aeneipennis*	<0.0001
T. ziegleri vs *X. aeneipennis*	<0.0001
T. angustula vs *X. aeneipennis*	<0.0001
T. fuscipennis vs *X. aeneipennis*	<0.0001

Table 2S. Critical thermal minima (CT_{Min}) and maxima (CT_{Max}), intertegular distance (ITD), head width (HW), and average ovary size (mm^2) among females of *Megalopta* with different reproductive status. Mean value is followed by SE and sample size.

**Reproductive status**	**CT_{Min} (˚C)**	**CT_{Max} (˚C)**	**ITD (mm)**	**HW (mm)**	**Ovary area (mm^2)**
Queen	10.69 ±0.22, *N* = 13	41.47 ±0.28, *N* = 12	2.84 ±0.09, *N* = 13	4.06 ±0.14, *N* = 13	3.08 ±0.34, *N* = 13
Worker	9.53 ±0.26, *N* = 17	41.29 ±0.26, *N* = 17	2.61 ±0.08, *N* = 17	3.64 ±0.11, *N* = 17	1.55 ±0.34, *N* = 17
Solitary reproductive	9.92 ±0.17, *N* = 40	41.39 ±0.25, *N* = 31	2.81 ±0.04, *N* = 40	3.98 ±0.06, *N* = 40	2.67 ±0.18, *N* = 40

Table 3S. Cox proportional hazards estimates of the survival of nocturnal and diurnal bees after exposure to a heat stress event (38 ˚C) over 5 hours. *P*-values refer to comparisons with nocturnal bees. Significant values in boldface. HR = Hazard ratio; CI = Confidence interval. See Table 7S for species and sample size used in this experiment.

**Bee**	**HR (95% CI)**	***P*-value**
Nocturnal bees	—	—
Orchid bees	1.15 (0.69–1.91)	0.6
Stingless bees	0.41 (0.22–0.78)	**<0.01**

Table 4S. *P*-values of pairwise comparisons among survival of nocturnal and diurnal bees using a Log-rank test after a heat stress event (38 ˚C) over 5 hours. Significant *P*-value in boldface. See Table 7S for species and sample size used in this experiment.

	Nocturnal bees	Orchid bees	Stingless bees
Nocturnal bees	—	0.49	**<0.01**
Orchid bees	0.49	—	**<0.01**
Stingless bees	**<0.01**	**<0.01**	

Table 5S. Survival time (hour) and percentage of water loss between nocturnal and diurnal bees exposed to a desiccant. Mean value is followed by SE and sample size. See Table 7S for species and sample size used in this experiment.

Treatment	Diurnal bees	Nocturnal bees
	Survival time (Hour)	
Control	14.77 ±2.20, *N* = 30	46.45 ±3.26, *N* = 27
Desiccant	14.87 ±1.47, *N* = 30	28.98 ±1.83, *N* = 31
	Water loss (%)	
Control	16.55 ±1.74, *N* = 30	21.88 ±2.63, *N* = 23
Desiccant	28.04 ±2.56, *N* = 30	22.35 ±1.77, *N* = 24

Table 6S. Results of pairwise comparisons with Bonferroni adjustment of the survival time and percentage of water loss of nocturnal and diurnal bees exposed to a desiccant. *DF* 1 in all comparisons. Significant *P*-value in boldface. See Table 7S for species and sample size used in this experiment.

**Comparison**	**Survival time**	**Water loss**
Control diurnal vs Treatment diurnal	1.000	**0.001**
Control diurnal vs Control nocturnal	**<0.001**	0.577
Control diurnal vs Treatment nocturnal	**0.003**	0.404
Treatment diurnal vs Control nocturnal	**<0.0001**	0.333
Treatment diurnal vs Treatment nocturnal	**0.006**	0.439
Control nocturnal vs Treatment nocturnal	**<0.0001**	1.000

Table 7S. Bee species used in the acute heat stress and desiccation stress assays (indicated with a plus sign). Number of individuals indicated in parentheses.

**Species**	**Acute heat stress**	**Desiccation stress**
Diurnal bees		
Euglossa bursigera Moure	+ (1)	
E. crassipunctata Moure	+ (2)	
E. igniventris Friese	+ (1)	
E. imperialis Cockerell	+ (11)	+ (2)
E. mixta Friese	+ (3)	
E. tridentata Moure	+ (4)	
Eulaema meriana (Olivier)	+ (15)	
Partamona orizabaensis (Strand)	+ (6)	+ (14)
Plebeia franki (Friese)	+ (4)	
Tetragona ziegleri (Friese)	+ (17)	+ (9)
Tetragonisca angustula (Latreille)	+ (1)	+ (15)
Trigona fulviventris		+ (7)
Trigona fuscipennis Friese		+ (13)
**Nocturnal bees**		
Megalopta amoena (Spinola)	+ (2)	+ (10)
M. genalis Meade-Waldo	+ (33)	+ (48)

Figure 1S. Temperature (a) and relative air humidity at the forest understory where nests of *Megalopta* are commonly found (~1 m above ground). Box plots show median, quartiles, and extreme values. For each figure, a different letter above bars indicates significant differences ($P < 0.05$).

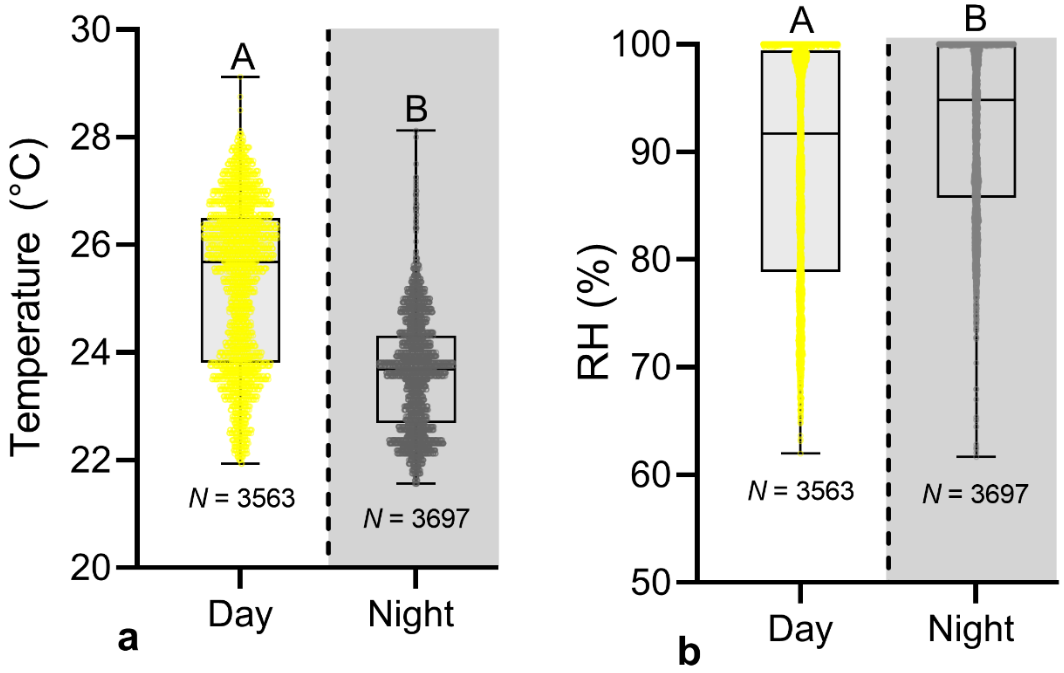

Figure 2S. Ambient and internal nest temperature of nocturnal sweat bees of the genus *Megalopta*. a) Box plots showing median, quartiles, and extreme values of temperatures. Groups with different letters are significantly different ($P < 0.05$). b) Changes in temperature at 5 min intervals during a 4 h period, from 9:30 to 13:30 h.

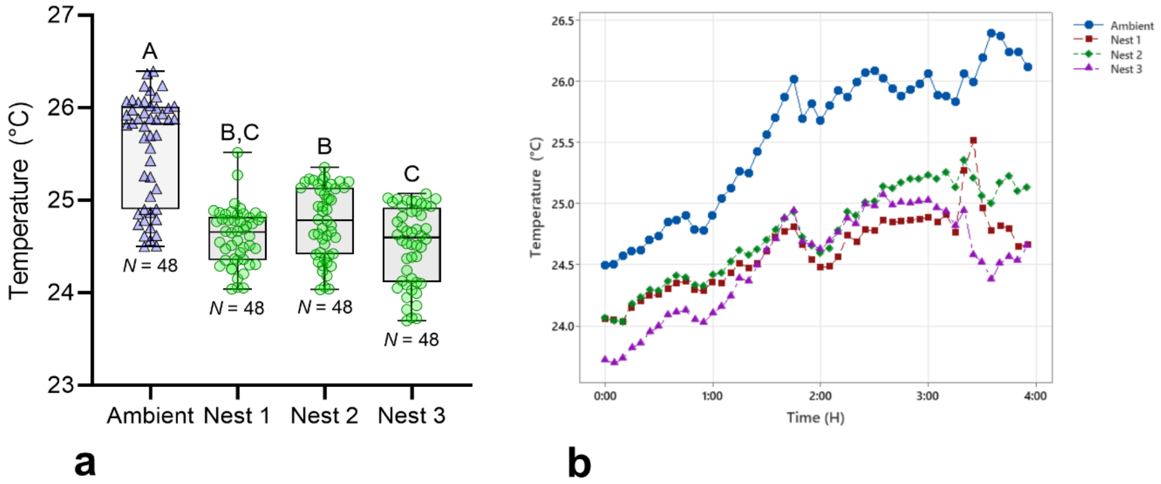

Figure 3S. Box plots showing CT_{Min} and CT_{Max} among species of diurnal and nocturnal bees. Species are organized into broader taxonomic groups (carpenter bees, orchid bees, stingless bees, and nocturnal bees). For each plot, a different capital letter above taxonomic group indicates significant differences ($P < 0.05$). To facilitate comparisons, a horizontal line was placed at 10 °C in the CT_{Min} plot and at 45 °C in the CT_{Max} plot.

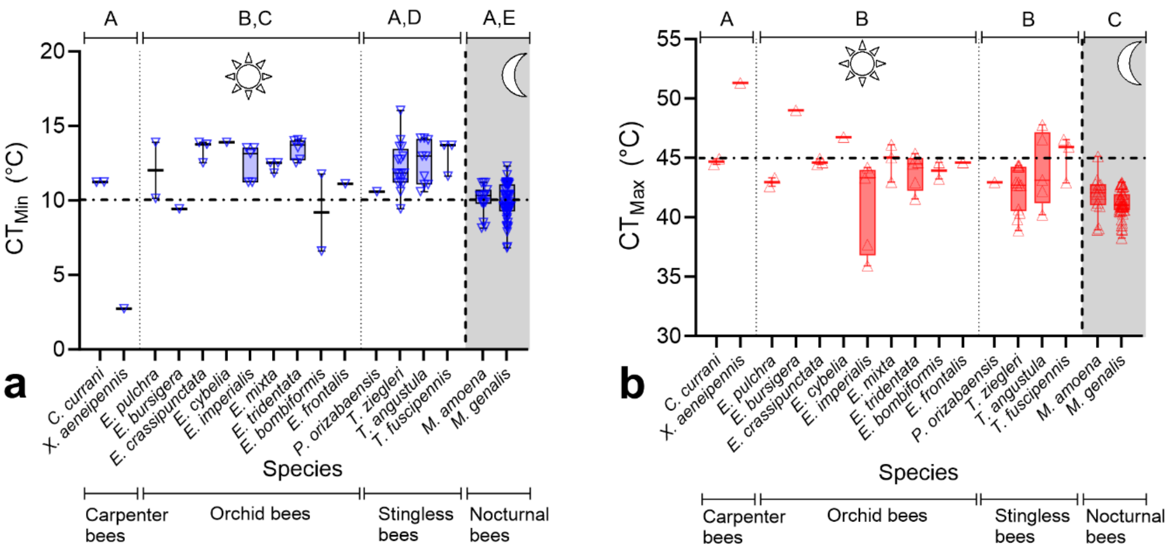

Figure 4S. Critical thermal minima (CT_{Min}) and maxima (CT_{Max}) and their relationship with maximum head width (HW) and average ovary area in nocturnal bees.

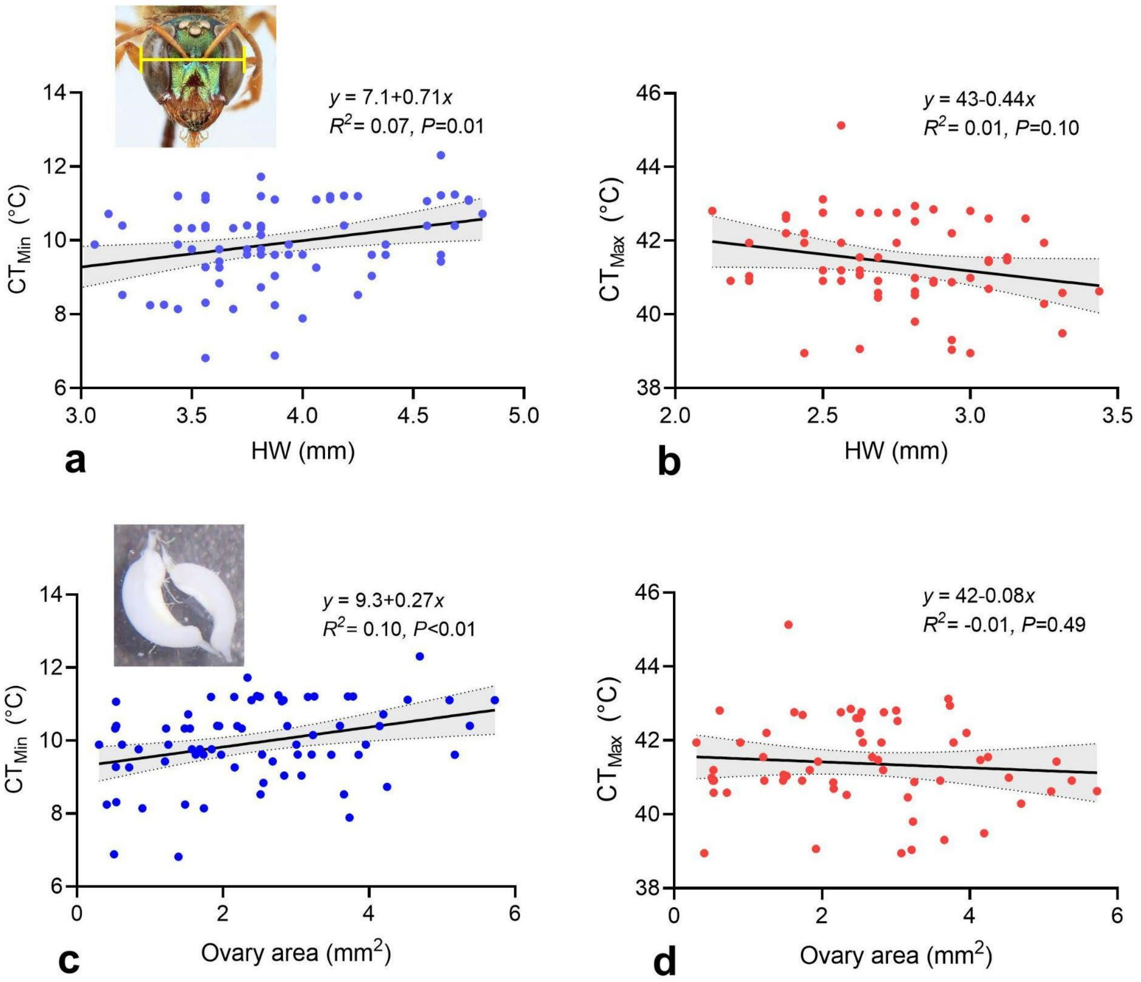

Figure 5S. Critical thermal minima (CT_{Min}) and maxima (CT_{Max}) among females of *Megalopta* with different reproductive status. Box plots show median, quartiles, and extreme values of temperatures. For each trait, groups with different letters are significantly different ($P < 0.05$).

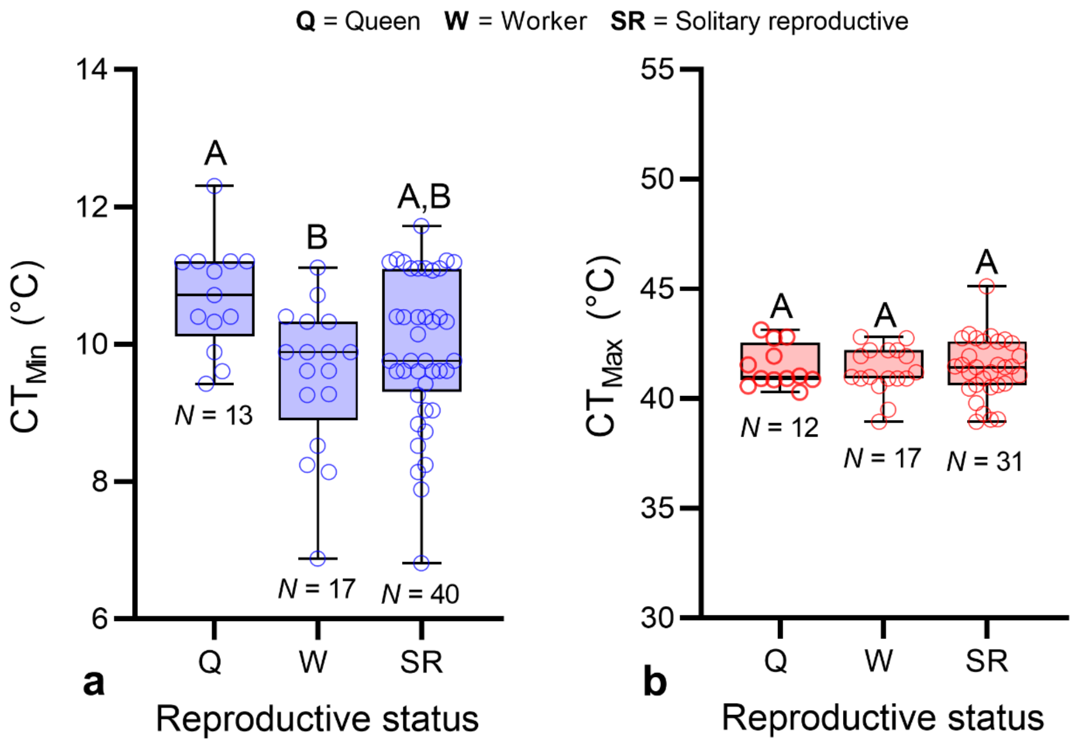

Figure 6S. Relationship between survival time of bees exposed to a desiccant and intertegular distance (ITD).

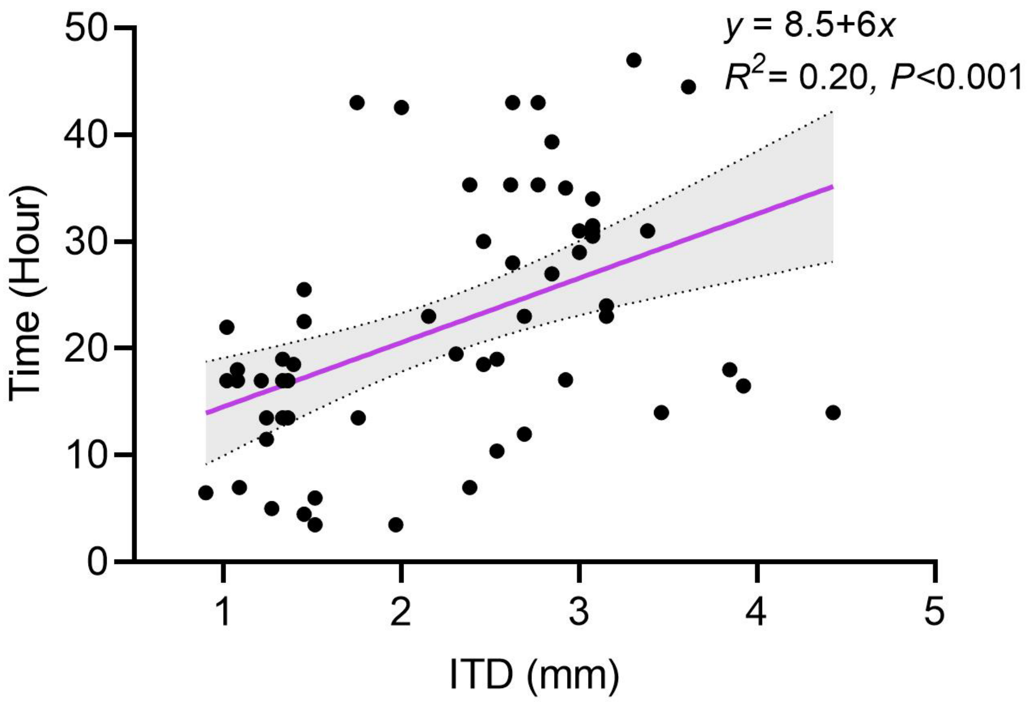

Fig 7S. Phylogenetic reconstruction of focal species. Node support indicated by the SH-aLRT value followed by the ultrafast bootstrap value after 1,000 replicates.

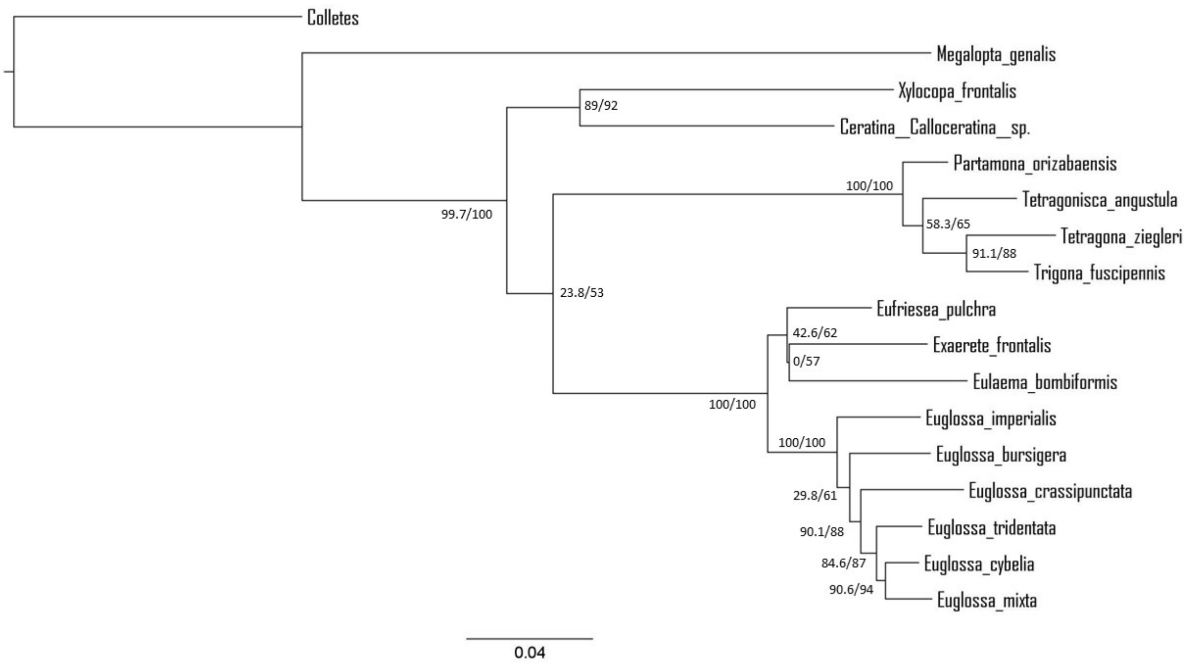

Fig 8S. Phylogenetic reconstruction of the focal bee species used in this study and their critical thermal minima (CT_{Min}).

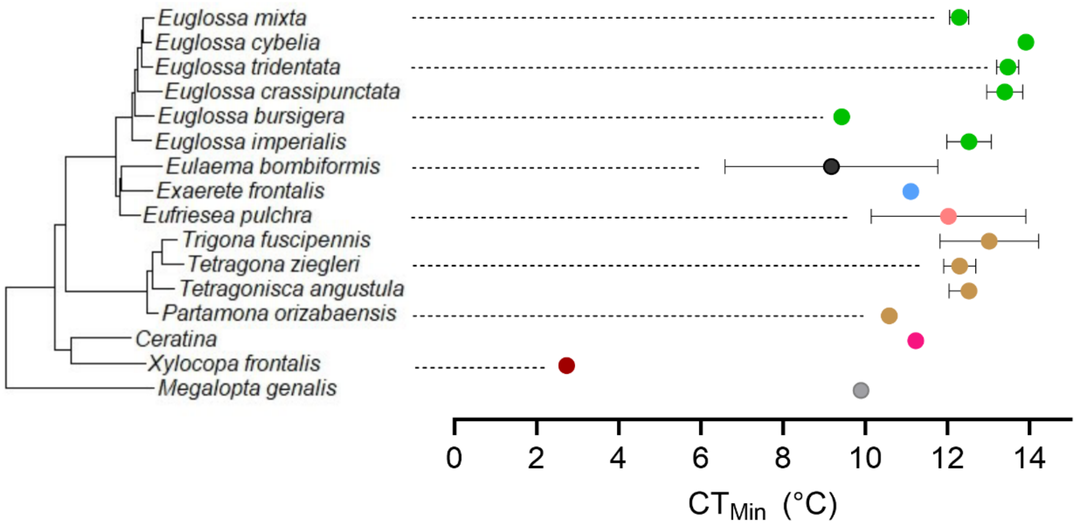

Figure 9S. Temperature and humidity sensor used in the field, thermal equipment, and desiccation apparatus. **a** = Thermochron Fob (red plastic holder) and iButton sensor. **b** = Plastic holder and iButton shielded with a piece of aluminum foil (~12 × 7 cm). **c** = Elara 2.0, a portable fully programmable heating/cooling anodized aluminum stage (outlined in red) designed for precision temperature control. Insert in upper right corner shows testing vials with bees, which are plugged with a moisten cotton ball. **d** = desiccation apparatus filled with fully dehydrated Drierite desiccant (treatment, left tubes) or with a moistened paper towel (control, right tubes).

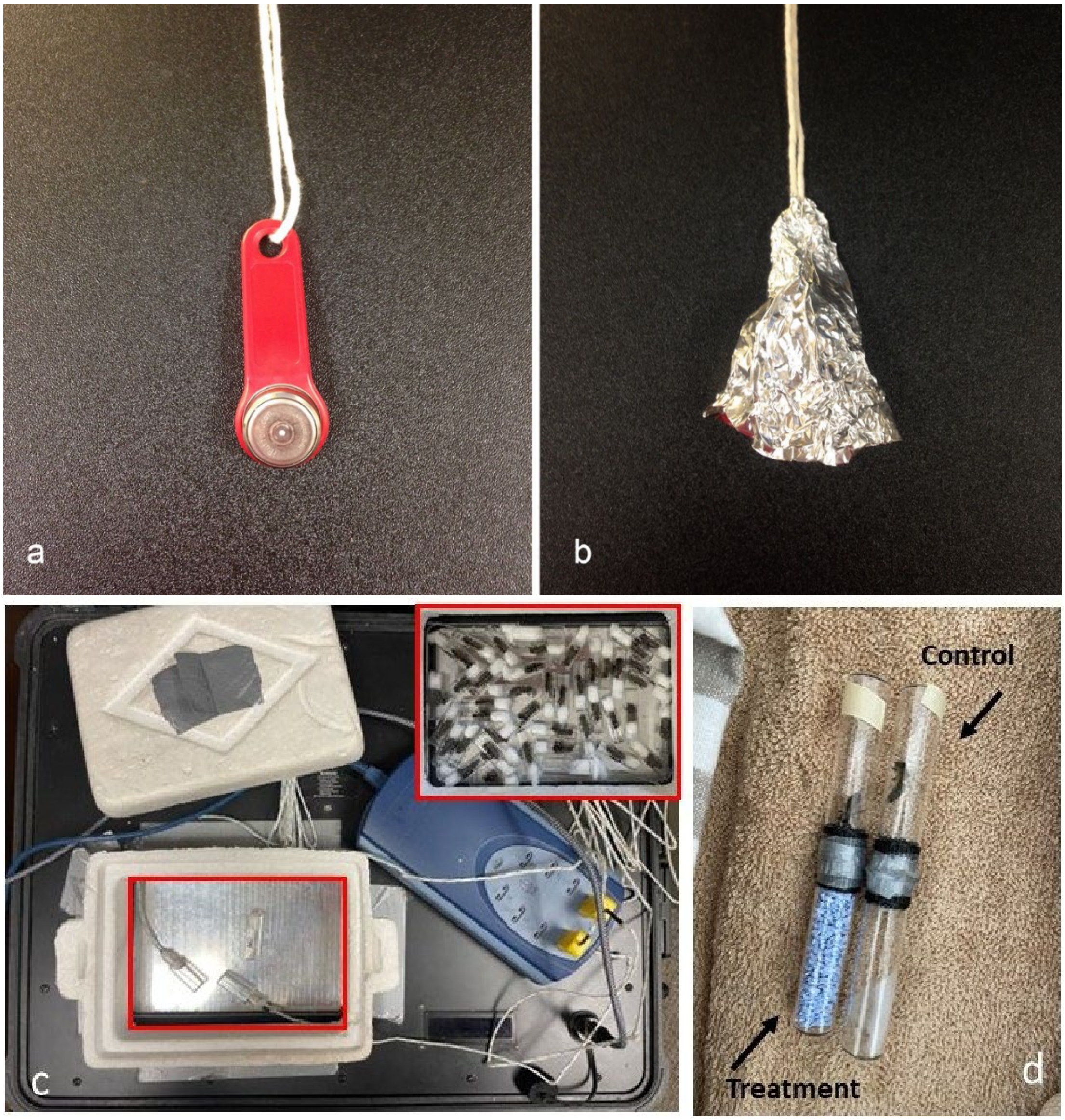

Supplementary methods

Phylogenetic inference. We used sequences available through GenBank[1] for the following five nuclear and mitochondrial gene fragments: arginine kinase (AK), elongation factor 1-α (EF1α), cytochrome oxidase I (COI), cytochrome b (CytB), and the 28S rDNA. We chose *Colletes inaequalis* Say as the outgroup. Ingroup sampling included sequences available for our focal species or their closest relative, as in the case of *X. aeneipennis*. We used Geneious prime® 2023.0.4 (http://www.geneious.com/) to align and concatenate sequences. We aligned sequences using the MAFFT Alignment option with default settings[2], with a final concatenated alignment length of 4,504 bp. We inferred trees with the Maximum likelihood (ML) software IQ-TREE[3] 1.6.12 through the IQ-TREE web server (http://iqtree.cibiv.univie.ac.at/). For ML inference we partitioned the concatenated dataset *a priori* by marker (*i.e*., each marker composing a single data subset). Substitution models were searched using the "Auto" option and the edge-proportional partition model. We assessed branch support with 1,000 Ultrafast bootstrap replicates[4] and single branch tests via SH-like approximate likelihood ration test (SH-aLRT[5]). Trees were visually inspected and annotated in FigTree v.1.4.4 (http://tree.bio.ed.ac.uk/).

References

1. Benson, D.A. *et al*. GenBank. *Nucleic Acids Res.* **41**, D36-D42 (2012).
2. Katoh, K. & Standley, D.M. MAFFT Multiple Sequence Alignment Software Version 7: Improvements in performance and usability. *Mol. Biol. Evol*. **30**, 772–780 (2013).
3. Nguyen L-T, Schmidt, H.A., von Haeseler, A. & Minh, B.Q. IQ-TREE: A fast and effective stochastic algorithm for estimating Maximum-likelihood phylogenies. *Mol. Biol. Evol*. **30**, 268–274 (2015).
4. Minh, B.Q., Nguyen, M.A.T. & Von Haeseler, A. Ultrafast approximation for phylogenetic bootstrap. *Mol. Biol. Evol*. **30**, 1188–1195 (2013).
5. Guindon, S. *et al*. New algorithms and methods to estimate maximum-likelihood phylogenies: assessing the performance of PhyML 3.0. *Syst. Biol.* **59**, 307–321 (2010).

Table 8S. GenBank accession numbers for sequences used in this study.

Species in this study	Species in Genebank	AK	EF1α	COI	CytB	28S
	C. inaequalis	—	AY585123	EF028488	—	AY654484
C. currani	*C.* (*Calloceratina*) sp.	—	GU321639	JX968036	GU321574	—
E. pulchra	*E. pulchra*	EU421637	EU421377	EU421506	—	HM750234
E. bursigera	*E. bursigera*	EU421701	EU421446	EU421573	AY916113	—
E. crassipunctata	*E. crassipunctata*	EU421626	EU421365	EU421494	AY916120	—
E. cybelia	*E. cybelia*	EU421645	EU421386	AY506430	AY916110	—
E. imperialis	*E. imperialis*	EU421668	EU421408	EU421537	AY916117	HM750232
E. mixta	*E. mixta*	—	EU421436	EU163094	AY916106	—
E. tridentata	*E. tridentata*	EU421702	EU421447	EU421574	AY916105	—
E. bombiformis	*E. bombiformis*	EU421655	EU421395	EU421524	AF002728	—
E. frontalis	*E. frontalis*	AY267175	AY267143	EU421478	AY916098	HM750233
P. orizabaensis	*P. orizabaensis*	FJ042241	FJ042342	—	—	FJ042066
T. ziegleri	*T. ziegleri*	—	FJ042387	AF066991	AF002725	FJ042115
T. angustula	*T. angustula*	—	FJ042381	MN344929	KF891236	FJ042109
T. fuscipennis	*T. fuscipennis*	EU184829	EU184770	—	—	HM750238
X. aeneipennis	*X. frontalis*	—	JQ230049	KC853310	AY005275	—
M. genalis	*M. genalis*	—	AF140316	JQ266453	—	JQ279190
